# Developing the language of futility in psychiatry with care

**DOI:** 10.4102/sajpsychiatry.v22i1.978

**Published:** 2016-10-24

**Authors:** Willie Pienaar

**Affiliations:** 1 Department of Psychiatry, Stellenbosch University, South Africa

## Abstract

In psychiatric practice, treatment success is, in many instances, not an achievable goal. Psychiatrists may often not acknowledge treatment failure in their patients and seldom consider that patients may be in situations that share similarities with end-of-life dilemmas in general somatic medicine. In such instances, futile treatment may be continued and patient suffering may be prolonged. Doctors should play a leading role in patient education, diagnosis, promoting best treatment options, motivation and support, but patients should be given the opportunity to take ownership of their illness and their future. In the discipline of psychiatry, physician-assisted suicide may be an option but warrants careful consideration. Contemporary psychiatrists may act paternalistically, refusing to accept the limitations of their scientific skills and/or struggle with the moral good of ‘letting go’ when required. It is arguably the seeming complexity of gauging patients’ understanding (competency, capacity) to make informed decisions that perpetuates futile treatment. Most patients, even in the presence of ongoing serious psychiatric illness, are able to give consent. Psychiatrists should be aware of the difference between *being alive* and *living*. Ongoing suffering cannot be condoned. The *personhood* of every patient and his/her bio-psycho-social and spiritual needs should, as far as possible, be respected. Psychiatrists should embrace the realisation of treatment futility and, in some cases, end-of-life decisions and take on the challenge as well as the responsibility of serving patients with mental illness in the best way possible.

## Introduction

### Case scenario

A patient of yours, now 58 years of age, suffers from difficult-to-treat bipolar disorder. Her illness started at 22 years of age with a depressive episode. She has a clear family history of bipolar illness. Despite her illness, she completed a master’s degree in economics, married at the age of 24 and has two daughters. The course of her illness has been progressive and difficult to treat, and she has suffered many relapses and numerous admissions, most of them as an involuntary inpatient. In the past 10 years, the duration of remission from illness has shortened considerably. She now receives a disability grant, is divorced for 20 years, has very little contact with her adult children, suffers disabling side effects of her medication, lives in a single room in an old-age home, has poor support systems and because of her illness has no friends. She has had the best treatment available without success. You have cared for the patient for the past 20 years and it pains you to know that treatment has failed. On her last visit, she was mildly depressed but voiced that her quality of life was poor and asked you for PAS (physician-assisted suicide). She said that she did not want to suffer any longer, did not want to be admitted to hospital again and indicated that she had made peace with death.

#### Are we as psychiatrists not living a paradigm far removed from the reality?

Contemporary debate on end-of-life decisions and the availability of physician-assisted dying for the few patients who may request such a service is rife, yet needed. Internationally, clear progress has been made and therapist-assisted death has become more readily available for a few selected and deserving cases. In South Africa, this complex moral debate is very much on the forefront, but as yet, little has been achieved. Work on future legislation on PAS has been developed and proposed but not promulgated. The current legal position is that PAS or active voluntary euthanasia is unlawful.^1^ On 30 April 2015, Judge Fabricius handed down a judgement in the High Court allowing advocate Robin Strasham-Ford to ask a doctor to help him end his life and also declaring that the doctor would not be acting illegally.^1,2^ Although the advocate had died before he could be assisted in this way, this ground-breaking judgement may pave the way to make it possible for a competent self-determining person, suffering the burden of an end-of-life disease, to die of own choice and with dignity.

The purpose of this paper is not to argue for or against PAS, but to encourage psychiatrists to acknowledge the reality of *futility* in psychiatric practice. This encouragement, however, should not be used as licence to decide prematurely that further interventions would be futile.

One dare say that, other than in neuropsychiatric disorders, psychiatry as a discipline does not often consider or contemplate end-of-life decisions. Psychiatric language does not include ‘failed treatment’ or ‘futile treatment’. Futility is seldom discussed in clinical and academic settings. Psychiatrists should reflect on the reason for this practice. Instead, we speak of ‘resistant to treatment’, ‘non-responder’ and ‘chronic disorder’. Yet as experienced psychiatrists, we cannot claim that we have never been faced with failed treatment or future *futile* treatment. Are psychiatrists trapped in a state of denial, or dare they not admit the futility of further intervention? All psychiatrists have, arguably, experienced a few patients with mental illness who could rightly be described as having a quality of existence so poor, so painful, that we should consider their existence to be at the end-of-living.^3^

A clear distinction should be made between the following two scenarios. One, further treatment becomes futile because life will end soon, caused by the natural progression of the illness. At the beginning of illness and for months and maybe years, treatment was effective, but the natural course of illness has made further treatment futile. Examples would be diabetes mellitus, cancer and Alzheimer’s disease. Past treatment was successful, but at some stage of the illness ongoing treatment may only add to the burden of illness. End of life becomes imminent. Two, treatment should be stopped (has become futile) because treatment failed (i.e. in treatment-resistant illness). Past treatment was only partially successful or treatment did not better the quality of life and the burden of treatment outweighs the burden of illness. The illness will continue, suffering will continue and ongoing treatment serves no purpose. Life and suffering (the case of a psychiatric illness) may continue for months and years. Examples include the following: resistant to treatment schizophrenia, bipolar disorder, anorexia nervosa, obsessive–compulsive disorder and others. Patients should be fully informed about anticipated future outcomes of ongoing treatment so as to empower them towards self-determination.

#### Is there a difference between living and being alive?

Can end-stage psychiatric illness (no benefit gained from further treatment) be a burden and can suffering in end-stage psychiatric illness be the same as in end-stage somatic illness? The reality is that life mostly ends in the context of a futile-to-treat somatic (physical) illness.^4^ The illness follows a natural course and ends suffering. This is not usually the case in a patient suffering a failed-to-treat psychiatric disorder. Life does not end then, suffering continues and the following should then be reflected on. Being alive and living is qualitatively not equal. Does being alive have moral value or does living have moral value? Living can be considered as a state that promises some quality of life that a patient experiences as worthwhile and as having more personal benefits than burden or disadvantage – a life that must continue as a personal choice.^5^

In the following paragraphs, the authors draw similarities between a number of end-of-life issues within the context of psychiatric practice.

### The doctor–patient contract

Clinicians are entrusted with making a diagnosis and psycho-educating their patients on diagnosis and appropriate management. They have a responsibility to evaluate treatment response over time and to provide ongoing guidance, motivation and support. As autonomous beings, patients should be encouraged to take ownership of their illness, ask questions, participate, decide, adhere to treatment and make necessary lifestyle changes. Therefore, within the doctor-patient contract, there are both separate and shared responsibilities. When best knowledge, skills and science fail patients and further treatment becomes futile, it is good clinical practice for both the clinician and patient to participate in planning for and choosing the road ahead.

#### What is truly worth doing in the face of futile treatment?

Clinicians who have a restricted understanding of what it means to care may believe that they should never abandon, never stop treatment or never let go. This imperative to intervene in the course of illness is a core characteristic of good care in most situations, even in the face of multiple obstacles, and may mistakenly be taken to constitute an obligation to never abandon treatment (even when futile) and thereby disallow patients their right to self-determination. Clinicians may possibly be afraid of facing their own failure, burnout or ‘futility’.^6,7^ In the event of truly failed treatment, the psychiatrist should have the courage to reflect on the unspoken doctor–patient contract in terms of what action would represent moral good.

### Current treatment outcomes in psychiatric practice

As with the rest of medicine, treatment resistance and treatment failure are realities in psychiatry. An estimated 30% – 60% of patients with schizophrenia do not respond adequately to conventional antipsychotic medication.^8^ After 10 years of the illness, 15% of patients with schizophrenia continue to be hospitalised and 10% die prematurely, mostly through suicide. After 30 years of being ill, 10% of patients are hospitalised and 15% are dead, mostly through suicide. Patients with chronic schizophrenia generally have a very poor quality of life.^9^

As many as 50% of patients treated with antidepressants do not achieve remission.^10^ Treatment-resistant depression (TRD) poses a major health problem in our society.^11^ In patients with ongoing TRD, the probability of recovery within 10 years is about 40%. TRD is associated with poor quality of life and increased mortality.^12^ Even with maintenance treatment and achievement of remission, studies indicate that many patients will suffer a relapse in the next year of treatment. The cumulative sustained recovery rate for patients with depression is 43%.^13^

Only about 60% of patients who suffer anxiety disorders respond significantly to treatment, with many patients turning out to be treatment-resistant. Patients who do not respond to treatment have a poor quality of life and have high rates of suicide.^14^ Clinical trials have shown that 40% – 70% of patients suffering from either generalised anxiety disorder, panic disorder or social anxiety disorder respond to treatment, 20% – 47% go into remission and about a third of patients do not respond.^15^

Patients suffering from anorexia nervosa have the highest mortality rate of any mental disorder and up to 50% meet criteria for a depressive disorder. Up to 20% of individuals with anorexia nervosa die prematurely from complications related to their illness.^16^ Approximately 20% of patients develop a chronic course with many years of suffering.^17^ Patients with eating disorders have elevated mortality rates and this is especially so with anorexia nervosa.^18^ Lopez et al., in reviewing a case of failed treatment in a patient suffering from anorexia nervosa, highlighted the complexity of palliative and hospice care in the face of futility.^19^

As important as the literature on treatment failure, are the anecdotal clinical experiences of failed treatment by clinicians. Even as clinicians celebrate the advances in science, treatment outcomes for many patients with mental disorders are poor. In the face of failed treatment, clinicians are often challenged with value decisions on what future action would benefit these patients best. Value decisions are those decisions made by building moral arguments that consider the values, wishes, needs, ideals and future plans of the patient. Value decisions cannot be argued by merely adding up (scientific) facts or clever wordplay (rhetoric) or driven by emotions. With value decisions, there may not be one correct answer. The therapist and patient may value the burden of illness, and therefore future management, differently. Within the clinician and patient relationship, the clinician should respect the burden ‘value’ the patient experiences. The clinician is motivated to act in the best interest of the patient. Why? Because the patient is ‘suffering’ or ‘enjoying’ the benefit of his/her final value decision and subsequent action that is taken.

### Treatment failure and futility

For the sake of a clear argument, one has to define ‘futility’ in clinical practice. In general medicine, futility of treatment is often discussed by the healthcare team. Should further treatment be futile (i.e. without expected benefit) and the end of life becomes imminent, palliative care seeks to focus on the quality of life for patients until the end of life. In our profession, care is accepted as morally and clinically good. Take a similar clinical situation in the discipline of psychiatry – the moral good action is much more complex to decide and act on.

Bernstein proposed the following conceptualisation of futility for clinicians in practice:

- **Physiologic futility:** There is no reasonable scientific evidence that the body could react physiologically towards recovery.
- **Imminent demise futility:** There may be a physiological response by the body, but the overwhelming end-stage medical condition cannot be reversed.
- **Clinical or overall futility:** Treatment may leave the patient alive but not able to interact meaningfully with the environment and with others. The patient is left with no quality of human existence.
- **Quality of life futility:** The final outcome of further treatment does not meet the desire, needs and goals of a person.^20^

These four ‘definitions’ of futility do not lie on a continuum. Rather, these are separate entities that pose difficult value decisions to end-of-life treatment teams.

Also, there is no commonly accepted community concept of treatment futility. Every person may have his/her own concept of treatment futility. Knowing this, the therapist should focus on the decisions, needs, choices, ideals and goals of the (informed) diseased individual. A question that arises is whether clinicians truly respect patient autonomy in these complex cases. The therapist should nurture his/her personal concept of treatment futility, respect the norms and standards of treatment outcomes by his/her profession, remind him/herself of community ideas on the matter, but take care to listen to and be motivated by the wishes, needs, ideals and future planning of his/her patient. In the end, ‘patient benefit’ and ‘patient best interest’ should pave the way to virtuous action.

On the other hand, as clinicians we are not mere agents to satisfy every request of our patients. Patients’ requests often fall outside of acceptable norms and standards, may not constitute good clinical action and may not represent the moral good. The norms and standards of clinical practice should represent the ‘best moral standard’. As our science evolves and the world changes, it may be time to reconsider the ‘moral good action’ when faced with futility-of-further-treatment cases.

Should our profession reconsider and change our stance on the issue of managing further futile treatment, then any new norms and standards should also be communicated to our patients.

### Suffering and personhood

Cassel wrote that suffering is an affliction in which the intactness of not only the body but also the self or person is threatened.^21^ Many aspects of personhood may be influenced. These include personal values, wishes, ideals, expressed emotions, social roles, sexuality, relationships, spirituality, future expectations and ‘being’. He asked, ‘How much of your personhood would you be willing to lose, before you would no longer want to continue suffering’.^21^ Dees and colleagues came to the following conclusion:

Unbearable suffering in the context of a request for PAS is a profoundly personal experience of an actual or perceived impending threat to the integrity or life of the person, which has a significant duration and a central place in the person’s mind.^22^

Clearly not all patients with a psychiatric illness who experience failed treatment are suffering to the extent that one could call it end-of-life suffering. Some patients do. Chronic schizophrenia, bipolar disorder, dementia, obsessive–compulsive disorder and others may cause suffering that most people would experience as end-of-life.

Ongoing personal suffering, even in the absence of physical illness, may be enough reason for some patients to ask for, or to consider, ending life. Clinicians should always be willing to remind themselves that these are the decisions of individuals and there is no common denominator for end-of-life decisions. Therefore, continued futile treatment should not be the choice of the clinician.

### Spirituality and end-of-life decisions

Psychiatrists, in their teaching and practice, are professionally bound to regard individuals as bio-psycho-social and spiritual beings. Most contemporary religions consider ending a life to be wrong. As clinicians, we celebrate diversity and we respect the spiritual beliefs of our clients. We should treat patients as ends in themselves, allowing and respecting the decisions of individuals as far as possible. Clinicians should not make hermeneutic decisions for their patients and should not explain and validate the interpretation of end-of-life decisions expounded by contemporary major religions. Clinicians should respect the patient’s personal validation of his/her religious beliefs as it extends to end-of-life decisions.

In general, ‘somatic’ healthcare practice clinicians, when facing future treatment futility, mostly respect and accept end-of-life decisions by patients. For example, many clinicians worldwide accept the ‘double effect’ of opiate-related pain treatment. In the case of ‘double effect’, death may be hastened by days or weeks in order to secure adequate pain relief. Early death is not intended but clinically accepted as best end-of-life care. Death may become a welcome relief and spiritually accepted by the individual patient. In contrast, psychiatrists do not have ‘morphine-pumps’ or other equivalent suffering-relieving options available for futile-to-treat psychiatric patients. Palliative care in psychiatry may be less successful in minimising suffering than in somatic medicine. This is all the more reason to respect the choices of patients by psychiatrists.

Experience has taught clinicians that patients nearing end-of-life may consider death in various ways. Patients may experience the nearing of death as ego-syntonic (i.e. they may welcome the end of suffering) or as ego-dystonic (i.e. an enemy and a terrifying experience). Some patients may be ambivalent about the approaching event. Emotions can vary from calm acceptance to anger, anxiety and depression. It may be best for an experienced clinician to ‘follow’ the patient, support the patient, provide information, share and deliberate on the best interests of the patient. If a well-informed, rational patient is suffering from a futile-to-treat psychiatric disorder and asks his/her therapist for assistance with ending life, the therapist should reconsider his/her existing beliefs/values on the matter and act in the best interest of the patient. Clinicians in the field of psychiatry should consider that letting go may, at times, be in the best interest of their patients.

### Living and being alive

As stated, when patients suffer a failure-to-treat mental disorder, the natural course of the disorder is not one of imminent patient death. Suffering may continue for many years. Continued suffering and the resulting quality of existence can only be truly judged and valued by the suffering person. In everyday clinical work, therapists do reflect on the seriousness of illness, but cannot in a subjective way validate the extent of suffering as experienced by the patient. Being alive does not imply living and ongoing suffering may be much worse than the benefits or positive effects of just being alive. The personal choice of patients is typically between suffering and living (not between suffering and being alive). Socrates said, ‘To fear death gentleman, is not only than to think oneself wise when one is not, to think one knows what one does not know. Death may be the greatest of all blessings for man, yet men fear it as if they knew it is the greatest of evils’. It stands to reason then that if there is no realistic chance of the suffering coming to end in the event of failed treatment, a patient’s choice should be facilitated in judging the quality of (not) ongoing life. Some rational beings will choose to end life and stop the suffering. Wittgenstein, the philosopher, said:

Death is not an event in life: we do not live to experience death. Death is non-existence; epistemologically there is no information about the prospect to be weighed alongside a continued, suffering-filled existence.^23^

In the past 20–30 years, the focus in healthcare has shifted from cure and maintaining life to ‘well-being’, as chosen by an individual.

When there is a failure of treatment in a patient suffering from an ongoing mental disorder and further treatment offers no benefit, the patient may experience the following: loss of control over one’s life, perceived sense of no meaningful future, the pointlessness of getting through the day, being a burden to others, unrelieved suffering, functional impairment, dependency, hopelessness, indignity, loss of autonomy and tired of life.^3,4,5,21^ Therefore, one can understand the ego-syntonic wish to end life in those patients who choose to end life because they are alive but not living.

### Who is competent in making an end-of-life decision?

In healthcare settings, informed consent is a Kantian ‘must’ respect rule. For informed consent, four specific abilities must be present in the individual from whom consent is sought: the ability to understand information about illness and treatment; the ability to understand how this information applies to their personal situation; the ability to reason with that information and the ability to make a choice and express such a choice (competency or capacity).^3,4,5,24,25^ In addition, voluntariness, no coercion, freedom to decide and the opportunity to build arguments towards personal choices must also be present. If all these qualities are present, the patient’s wishes should be respected.

In everyday practice, psychiatrists are confronted with severely depressed patients with suicidal ideation. This is such a common occurrence that the action taken by the psychiatrist may become behaviourally paternalistic and reflect an institutionalised culture. The clinician may argue that if the illness can be treated and the patient has poor understanding (poor judgement on account of the illness), the action taken should be motivated not by patient competency or patient incompetency, but on the basis of what is known in terms of treatment outcomes – if an illness can be reversed and/or if suffering can be terminated. What happens if the best treatment truly fails and further treatment is futile (viz. the illness is not reversible) and suffering cannot be stopped? The irreversible mental disorder now becomes the irreversible ‘cancer ‘of somatic medicine, without the possibility of relieving suffering through palliative care.^5^ Experience has taught the clinician that the best bio-psycho-social palliative care in these patients does not end suffering. The clinician can continue (futile) treatment, because the patient may be judged as to be incompetent to make further treatment decisions. Stopping treatment may be seen as abandoning the patient. Treatment outcomes and patient best interest should motivate ongoing treatment, not patient competence or incompetence.

In decision making, clinicians must consider and balance, inter alia, the following: one, the level of competency of the patient (high or low); two, the weight and complexity of the decision (balancing possible outcomes and weighing the complexity versus the simplicity of decisions to be made by the patient); and three, the time factor – emergency or urgent decision, or a decision that can wait until patient competency has been restored (if possible).

In the *Principles of Biomedical Ethics* (Chapter 3) by Beauchamp and Childress, the levels of competency can be summarised as follows:^25^

- Inability to communicate a preference or choice.
- Inability to understand one’s immediate situation and its consequences.
- Inability to understand relevant information.
- Inability to reason.
- Inability to rational reasoning.
- Inability to risk/benefit reasoning.
- Inability to reach a reasonable person’s decision.

In considering the weight of the decision to be made, it is necessary that a patient have a competency level of least 5–6 to make an autonomous end-of-life decision.^25^

The decision to be made by the patient is not complex. The concrete understanding of futility is ‘simple’, factual, clearly communicated by the treating clinician or treatment team and experienced by the suffering patient. For the patient, what follows this factual information is the need to make complex and painful decisions relating to the future. Clinicians should be available to those patients who would like to discuss and who struggle with above end-of-life issues. What could be more concrete and simple than ‘This illness will not go away, I am suffering, and it is my choice to stop or to continue with my suffering’? This decision does not rest on complex arguments and understanding. The facts are simple and the painful decision should be viewed as a free choice to be made by a suffering person with the help of his/her therapist. This therapeutic process (support) should become part and parcel of this language.

A depressed mood state can compromise patient competency.^26,27^ However, not all severely depressed patients are incompetent to such a degree that they cannot make an informed decision. In fact, most depressed patients can make an informed decision if the decision to be made is simple and clear. If a depressed patient is automatically deemed incompetent to make an end-of-life decision, the treating clinician is unlikely to consider an end-of-life request made by the suffering patient. Clinicians need to evaluate every patient on a case-by-case basis. The practice of casuistry requires consideration of the essence of the situation, getting all relevant information, weighing and balancing all facts and outcomes, and then drawing from experience before making a decision on what the moral good action would be. If the illness is irreversible and suffering prolonged, the only alternative may be to end life – a simple decision that should be respected even if a depressed patient’s competency is mildly compromised by being depressed. If a patient understands relevant illness-related information, the futility of treatment and the meaning of this information to his own future, he should be allowed to make a personal choice that reflects his own ideals, goals and life philosophy. To date, there is no evidence that depression *per se* renders patients incompetent. Schuklenk and Van de Vathorst stated that, despite substantial research efforts, it is not possible to demonstrate that patients with depression are incompetent at evaluating their quality of life and future life prospects.^5^ Therefore, clinicians may need to come to grips with their imperfect science and not hide behind patient ‘incompetency’. Arguably, the majority of patients suffering from failed-to-treat mental disorders are competent enough to make and request end-of-life help. Patient competency is on a continuum and all possible outcomes should be weighed up, paying particular respect to patient autonomy.

A further aspect of patient decision making that needs to be accounted for is the expressed wishes, goals, ideals and personal philosophy prior to the onset of illness and in the face of ongoing suffering. The act and experience of living is shaped by past and present experiences of living and the prospect of quality of life in the future. Family and friends may be helpful in evaluating the premorbid attitudes and wishes of a patient with a compromised level of competency. Where competency is severely compromised, surrogate decision makers have to take into account the premorbid wishes, ideals and the expressed needs of the patient.

## Challenging obstacles and difficulties

If clinicians become part of end-of-life decisions and participate in helping those few patients who ask for PAS, will it not lead to the slippery slope phenomenon? Dembo in his article ‘Addressing treatment futility and assisted suicide in psychiatry’ identified the following potential problems:^3^

- A patient may lose autonomy through family and clinician coercion associated with caregiver burnout, family suffering and financial difficulties.
- It may prevent future development of improved standards of care.
- It may perpetuate ongoing stigma towards patients with psychiatric disorders and limit social support.
- Some psychiatrists may be biased towards PAS.
- All clinicians want to see the end of patient suffering.
- Offering PAS may reinforce the loss of hope for the patient.
- While these may be issues of concern, they cannot negate the fact that there are deserving patients.

Another dilemma that may arise is when a patient deserving of PAS has family members who oppose the patient’s request. This may be on account of ‘compassion’, family who are poorly informed, religious reasons and other reasons that benefit the family and not the patient.

In the case of ‘deserving’ patients who are under 18 years of age, as often is the case with anorexia nervosa, the moral debate on end-of-life decisions becomes more complex. Young lives are highly valued. Again, all arguments for stopping futile treatment, ending ongoing suffering and respecting autonomy, even at this young age, cannot be ignored. To know what is truly worth doing in life is at the core of this moral debate.

## Guidelines for physician-assisted suicide

Schuklenk and Van de Vathorst made the following suggestions concerning patients with TRD:^5^

- The patient is competent to evaluate their current situation.
- The patient is competent to evaluate their future prospects based on the scientific evidence available at the time.
- The patient is fully informed and can make a voluntary decision.
- The patient’s quality of life is such that they do not consider it worth living, and the likelihood of improving is exceedingly small or non-existent.
- The patient repeats his requests over a reasonable period of time.

In addition, the treatment team or the independent clinician (not the sole treating doctor) should establish that the best contemporary treatment options have been exhausted and that further treatment would be futile.

Internationally, services for PAS are not readily available. The Netherlands and Belgium, in Europe, and Oregon, Washington, Vermont, Montana and New Mexico, in the USA, have legalised end-of-life services.^28^ The Netherlands was the first to allow patients suffering from mental disorders to be allowed PAS based on strict criteria. How often do patients with psychiatric disorders ask their clinicians to help them make an end to their suffering? Snijdewind et al. reports in a study done in an end-of-life clinic for physician-assisted dying in the Netherlands, after 1 year of experience, that of the 645 patient requests only 25.1% were granted, 46.5% were refused, 19.2% of patients died before evaluation and 9.1% withdrew their request.^29^ Patients with somatic illness (32.8%) and cognitive decline (37.5%) had the highest percentage of granted requests. Only 18.7% of requests were from patients suffering from psychological disorders of which the lowest percentage (5%) was granted. The author acknowledges that PAS for psychiatric reasons is still controversial in the Netherlands and that two-thirds of physicians find it inconceivable to assist patients with psychiatric disorders. Failed requests in this group may deter deserving patients applying for PAS.^29^ Patients who suffer from a mental disorder only, with no co-morbid somatic disorder, can apply for PAS in European countries under stringent guidelines. The actual number of applications is low.^22^

The lead author, after 30 years of psychiatric practice in a government psychiatric hospital, managing difficult-to-treat clients in the community, reports that requests for PAS by deserving cases is extremely rare. Even if ‘rare’, the clinician, if confronted with an end-of-life request, should be able and willing to truly rethink and reconsider patient best interest. This experience may be generalised to most psychiatrists treating patients with serious psychiatric disorders in the government system. This fact cannot serve as an argument against the need for such a service. Should an appropriate, well-managed service be available, it may help psychiatrists do what is in the best interest of their patients. Despite the fact that there are few patients asking for PAS, the psychiatric profession should seriously contemplate the need for such a service that will serve to broaden management options.

## Conclusion

Clinicians have come a long way. From supporters and comforters of the ill, they have advanced to successful healers of the suffering. The essence of healthcare is targeted at patient welfare as decided by the user. Psychiatrists, too, have to face the limitations of scientific knowledge and the reality that treatment may be futile treatment in some instances. In psychiatric practice, if best treatment has failed, the prospect of further treatment futility should be acknowledged. Patients should be fully informed, all patient and family questions should be answered and clinicians should consider ‘letting go’ may truly be in the best interest of the patient. Both somatic and psychiatric illness end-of-life decisions may compromise competency. The facts that constitute futility are clearly understood by most somatic and psychiatrically ill patients. They should be helped with their complex and painful decisions about the future, acknowledging the full spectrum of possible end-of-life decisions for different patients. Arguments for and against PAS have been discussed. After weighing up and balancing the facts, values and arguments, the authors are in favour of PAS for those few deserving patients who choose this decision. Clear guidelines for PAS are needed. In the South African context, legislation on PAS has yet to get off the ground, despite tremendous legal and advocacy efforts that have been made in this regard. Clinicians should acknowledge the concept of futility in their everyday language and take the lead in raising awareness among patients, the community and the government of the day.
